# The Malawi National Tuberculosis Programme: an equity analysis

**DOI:** 10.1186/1475-9276-6-24

**Published:** 2007-12-31

**Authors:** Bertha Nhlema Simwaka, George Bello, Hastings Banda, Rhehab Chimzizi, Bertel SB Squire, Sally J Theobald

**Affiliations:** 1 Research for Equity And Community Health Trust, Area 3 Mtunthama Drive, Lilongwe, Malawi; 2 Community Health Sciences Unit, Ministry of Health, Mtunthama Drive, Area 3, Lilongwe, Malawi; 3 The National TB Control Programme, Ministry of Health, Mtunthama Drive, Area 3, Lilongwe, Malawi; 4 Liverpool School of Tropical Medicine, Pembroke Place, L3 5QA, UK

## Abstract

**Background:**

Until 2005, the Malawi National Tuberculosis Control Programme had been implemented as a vertical programme. Working within the Sector Wide Approach (SWAp) provides a new environment and new opportunities for monitoring the equity performance of the programme. This paper synthesizes what is known on equity and TB in Malawi and highlights areas for further action and advocacy.

**Methods:**

A synthesis of a wide range of published and unpublished reports and studies using a variety of methodological approaches was undertaken and complemented by additional analysis of routine data on access to TB services. The analysis and recommendations were developed, through consultation with key stakeholders in Malawi and a review of the international literature.

**Results:**

The lack of a prevalence survey severely limits the epidemiological knowledge base on TB and vulnerability. TB cases have increased rapidly from 5,334 in 1985 to 28,000 in 2006. This increase has been attributed to HIV/AIDS; 77% of TB patients are HIV positive. The age/gender breakdown of TB notification cases mirrors the HIV epidemic with higher rates amongst younger women and older men. The WHO estimates that only 48% of TB cases are detected in Malawi. The complexity of TB diagnosis requires repeated visits, long queues, and delays in sending results. This reduces poor women and men's ability to access and adhere to services. The costs of seeking TB care are high for poor women and men – up to 240% of monthly income as compared to 126% of monthly income for the non-poor. The TB Control Programme has attempted to increase access to TB services for vulnerable groups through community outreach activities, decentralising DOT and linking with HIV services.

**Conclusion:**

The Programme of Work which is being delivered through the SWAp is a good opportunity to enhance equity and pro-poor health services. The major challenge is to increase case detection, especially amongst the poor, where we assume most 'missing cases' are to be found. In addition, the Programme needs a prevalence survey which will enable thorough equity monitoring and the development of responsive interventions to promote service access amongst 'missing' women, men, boys and girls.

## Background

Through a consultative process, Malawi's Ministry of Health (MoH) has developed the Programme of Work (PoW) which outlines the processes through which to deliver the Essential Health Package (EHP) [1]. The development and implementation of the EHP was adopted as the sector's main pro-poor strategy and contribution to the Malawi Poverty Reduction Strategy Papers (MPRSP) [2]. The primary goal of the EHP is to ensure that health services are accessible to all Malawians. The rationale behind the EHP as a pro-poor strategy is to focus on the major causes of morbidity and mortality, and address medical conditions and service gaps that disproportionately affect the rural poor. The MoH has adopted the Sector Wide Approach to health development as the overarching strategy for the implementation of the PoW. The PoW outlines health activities to be implemented by MoH, development partners and major not-for-profit NGOs like CHAM (Christian Health Association of Malawi) [1].

The National Tuberculosis Programme has historically been implemented as a vertical programme and has implemented the WHO recommended Directly Observed Treatment Short Course (DOTS) strategy since 1964. The DOTS strategy has five elements; government commitment, case detection through passive case finding, administration of standardised short course chemotherapy to at least all confirmed sputum smear positive cases of tuberculosis under proper management conditions; establishment of system of regular drug supply; and establishment and maintenance of a monitoring system[3].

In the first half of 2005, in response to the development of the PoW the Malawian National Tuberculosis Control Programme began the process moving away from a vertical programme and realigning its planning, approach and budgeting to be in line with the SWAp.

The Research for Equity and Community Health (REACH) Trust was commissioned by the Health Sector's Sub Group on Equity and Access to carry out a synthesis study to feed into the baseline analysis for monitoring equity in the Health Sector SWAp, in line with the Programme of Work. The objectives of the synthesis study were:

1. To conduct a broad analysis of equity concerns in TB control programming in Malawi

2. To analyse evidence on different group's vulnerabilities to tuberculosis

3. To assess and correlate information on pathways and care seeking patterns of patients and the impact of tuberculosis on different socio-economic groups

4. Develop practical recommendations for the TB programme and the broader health sector in Malawi.

## Methods

A synthesis of existing evidence on equity and access to TB services in Malawi was undertaken to draw baseline information for monitoring programme performance. This synthesis was undertaken by the following:

1) Searching existing published and unpublished reports on access to TB services in Malawi. This involved searching national and international databases and contacting research institutions and the Ministry of Health. Data using a variety of methodological approaches were sought.

2) Conducting additional analysis: analysis of routinely collected data on service access from 1999 to 2005 by district and gender

3) Reviewing the international literature

4) Consultations with stakeholders including researchers and policy makers on availability of information, implications of the analysis undertaken and recommendations.

**Figure 1:**
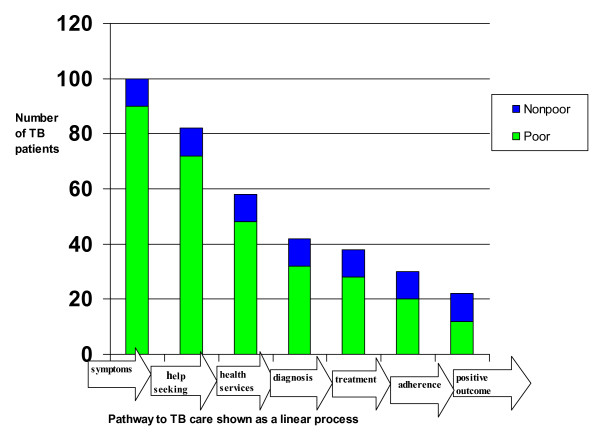
Pathway to TB diagnosis.

The analysis of the evidence collated was undertaken using the TB pathway to care. This pathway illustrated by figure 1: Pathway to TB care is an adaptation of modeling of numbers presented by Uplekar et al in 2001 which highlights stages of illness and aims at estimating the number of people suffering from disease to those who access health services, get a diagnosis, reach the treatment stage and achieve a positive outcome [4]. Gender and poverty analysis was applied. This is because an individual's journey through the pathway to care is shaped by different axes of vulnerability such as gender and poverty and how these are experienced within households, communities and health systems. Gender analysis is used to unpack how socially and culturally constructed behaviour, roles, expectation and responsibilities all women and men learn in the context of their own societies affects access and adherence to TB treatment. Poverty analysis is also deployed to understand how deprivation of income or basic needs, absence of infrastructure, can shape access to care and health outcomes [5].

## Findings

The findings are synthesized under the following themes: (1) epidemiological data linking TB and vulnerability, (2) pathways to care seeking, including health service related barriers, patient related barriers and the impact of care seeking on households (3) National TB Control Programme responses to equity challenges.

### Epidemiological data linking TB and vulnerability

There has been no population based prevalence survey on TB in Malawi. This severely limits what is known about TB and vulnerability, as only case notification data is available through passive case finding. That is data for patients who have successfully entered the formal health services, been confirmed as smear positive during the diagnostic process, and started on treatment, and hence included in notification rates. Figure 1, based on an analysis of global literature on TB and poverty, illustrates how cases can be lost through the pathway to care, and how poorer groups can be over-represented amongst these missing cases. In Malawi, the WHO estimates that only 48% of TB cases are detected in Malawi, meaning that a startling 52% of people with TB are 'missing' from TB services and hence TB notification rates [6]. This section presents what is known from population specific prevalence surveys that have been conducted and from analysis of case notification data. It then gives an overview of poverty and HIV prevalence in Malawi to illustrate the context in which actual and potential patients live.

Population specific prevalence surveys have been conducted among specific vulnerable groups such as prisoners and households of index TB patients. Both of these show higher prevalence rates of TB than that predicted amongst the general population. A study conducted in 1996 revealed that the prevalence rate was 5% among prisoners and 4.5% among prison staff [7]. This translated into an annual notification rate for 2000 of 4,478 per 100,000 as compared to an estimated 183 per 100,000 for the general population. A survey conducted to assess prevalence of TB was undertaken amongst households with smear positive patients in 22 districts. It was found that within these households 1.7% had a member who had developed TB as compared to 0.19% (p = 0.01) of the control group (households of patients without TB) [8].

The National TB Data show that TB notification has increased rapidly from 5,334 in 1985 to over 28,000 in 2006. The increase that has been attributed to HIV and AIDS, which according to a study conducted in – 77% of TB patients are HIV positive[9]. Women between 14 to 24 years of age are 4 times at risk of contracting HIV/AIDS than other groups[10], and given high levels of co-infection it is likely that the proportion of women in this age group who contract TB will also rise. Notification data show that district ratios of male to female notification rates vary and ranges from 0.75 to 1.55 and at national level it is almost equal [11]. However, from a national perspective it can be seen that more women then men are being diagnosed with TB in the 15–34 year age group and that this situation reverses in the over 35 year age group. This probably reflects higher HIV prevalence rates amongst younger women, and hence increased vulnerability to TB infections. There are also important regional variations in TB notification rates. The NTP routine data indicates that 50% of notified TB cases are from the urban cities of Blantyre, Lilongwe, Zomba and Mzuzu [11]. This means that 50% of TB cases at the moment are coming from about 20% of the Malawi population. This likely indicates less access to services in rural areas but could reflect higher TB incidence in urban areas.

**Figure 2:**
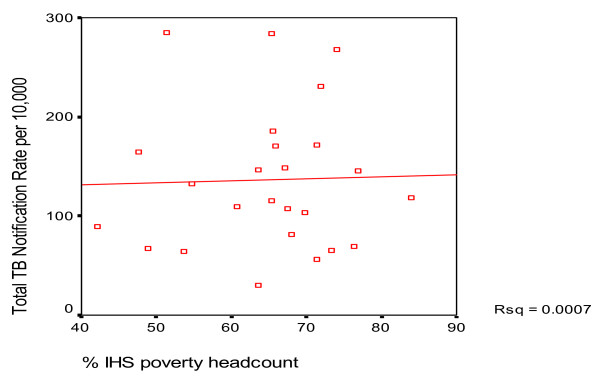
Poverty and TB notification.

**Figure 3:**
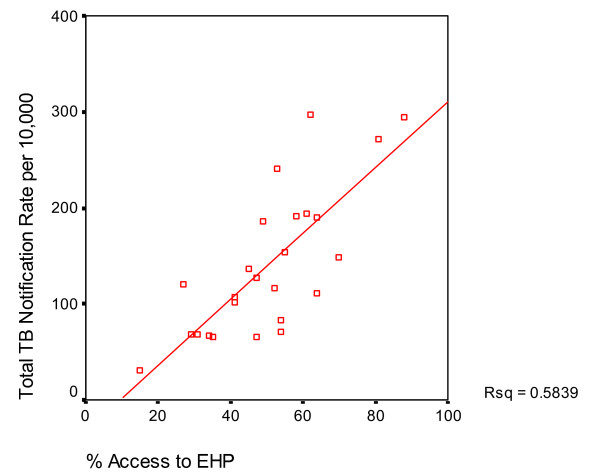
TB notification and Essential Health Package.

An equity analysis of routine data from1998 to 2004 for all Malawian districts highlights a lower utilisation of TB services by populations with limited access to health facilities [11]. The purpose of the analysis was to assess correlation between poverty levels and TB notification rates. The assumption was that District-specific TB notification rates are negatively correlated with the district-specific proportion of the population classified as poor, in other words amongst poorer districts there is likely to be lower TB notification rates. The assessment revealed no significant association between TB notification rates and poverty (overall r = 0.007, p = 0.97; men r = -0.1 p = 0.635; female r = 0.33 p = 0.87) (Figure 2: Scatter diagram for poverty levels and notifications for all districts). There are two possible explanations for the findings either TB incidence is not yet associated with poverty (possibly because the impact of HIV is dominant- see below) or the poor patients have less access to TB services than non-poor patients. However, these results need to be interpreted with caution due to the lack of information on community TB prevalence. However, there was a significantly positive correlation between TB notification rates and HIV prevalence (r = 0.657, p < 0.0001). The association existed even after adjusting for TB rates by sex (r = .542, p = 0.005 for male and r = 0.658, p < 0.0001 for females), poverty headcount and health facility coverage. Similarly, a significant positive correlation existed between TB notification rates and percentage of the population with access to Essential Health Package (EHP) (r = 0.764, p < 0.0001), even after adjusting the covariates assessed and has been shown by figure 3 (Correlation between TB notification rates and % with access to EHP). Thus districts with high HIV prevalence and good access to EHP had higher TB notification rates of tuberculosis.

Malawi, like many countries in Sub-Saharan Africa has high levels of poverty which affects both vulnerability to TB and access to health services, and helps to explain why many TB cases are missing from notification data. According National Statistics Office the poverty level in Malawi is 52.4% and higher in rural areas (55.9%) as compared to urban areas (25.4%)[12]. Malawi's poor are not a homogeneous group but consist of a cross-section of the population, including smallholder farmers with less than one hectare of land, estate tenants, the urban poor and female-headed households. The HIV/AIDS epidemic is further intensifying and shaping poverty in Malawi as well as increasing vulnerability to TB. The next section synthesises what is known about pathways to care seeking and the challenges this poses for poor women, men, girls and boys, and hence sheds light on why 52% of TB cases are missing from Malawian notification data.

### Pathways to care seeking

The pathway for care seeking is associated with people seeking a range of remedies from a variety of health providers at all stages of their illness [13-15].

At each stage there are barriers to progressing to the next stage. Consequently there will be a loss or 'drop-out' from the total number of people with symptoms, to those who are successfully treated for TB. Barriers which prevent people from successfully moving through this pathway are health service related, and also affected by an interplay of poverty, gender, geography and socio-cultural factors.

### Health service related barriers

**Figure 4:**
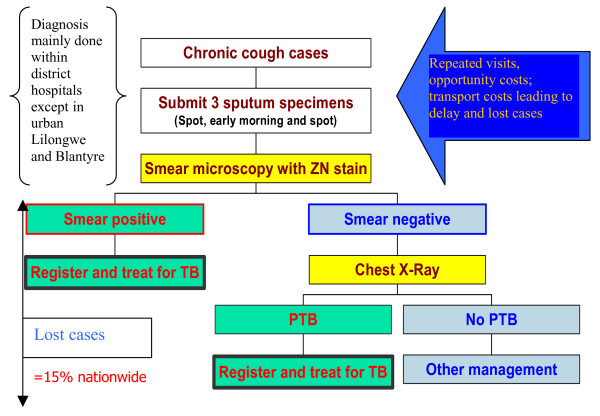
Steps to TB diagnosis.

Figure 4 (Steps to TB diagnosis) also illustrates the complexity of TB diagnosis requires repeated visits to health facilities and long queues. In Malawi studies conducted have shown that there are delays in transportation of specimens and communication of results which affects and reduces poor women and men's ability to access and adhere to services [15]. The situation in rural Malawi is more complicated and is likely to result in further drop-outs. Diagnosis for TB in rural districts is still centralised at the district hospitals and only treatment is decentralised to the rural health facilities. This approach to diagnosis entails sputum being sent to the hospital or patients going to the district hospital for diagnosis or x-ray. Studies conducted on pathways to care in rural districts have revealed that due to resource constraints, sending results back to health facilities is a complicated process and diagnosis of TB can take more than one month [15]. A study from Ntcheu District revealed that structural barriers related to the health care system were key to explaining extended pathways to diagnosis including delays in receipt of sputum results, along with the misconception that negative smears excluded the diagnosis of tuberculosis [15]. Typical quotes from qualitative research with patients/patients' families illustrating these structural barriers are as follows:

"We came back to wait for the result, we waited and waited but the result never comes out. We went to the health centre to find out about the result only to be told that the sputum was sent to Ntcheu District hospital, if the result came we will inform you. We waited and waited then we went again for a second time, third time only to be told we shall be called. We stayed at home....."

"She used to go to the health centre thinking they could help but there was nothing they could find...she was sure that she had TB....but the hospital was saying that they could not find TB, so she was sure that she had TB only the hospital was failing to do their job...they did the sputum examination but they told us she had no TB"

### Patient related barriers

In Malawi, as in other resource poor contexts, poor people depend on low income earning livelihood strategies. For example within urban settings, most poor people do *ganyu *work (daily employment), petty trading and in extreme cases begging to earn a living [16]. This has two consequences: a) it leaves the poor with limited disposable income to use for care seeking, b) it means that the poor have to choose to lose a day's earnings if they need to take a day off to seek health care. The situation is similar in rural areas; most of the poor depend on *ganyu *work or tend fields to earn a living [17]. Poverty therefore affects one's ability to access services due to the necessary expenditure on transport and food costs for patient and guardians alike, despite TB services and drugs being free. Evidence available in Malawi indicates that the total delay for TB patients before diagnosis is up to 2 months [18]. In Malawi, studies have revealed that in urban areas, patients with symptoms suggestive of TB exhibit a variety of initial care-seeking patterns but most are characterised by repeated visits to some combination of informal providers such as local storekeepers. Care seeking from public health facilities tend to occur more as symptoms progress [19]. Poor patients in particular are likely to go first to the informal private sector where they face fewer costs and opportunity costs, and they tend to stay at this level longer than non-poor patients. Once within the formal health system, patients need to make several visits due to the requirements for sputum submission for diagnosis. The longer pathways undertaken by the poor are given some credence by the Geographical Information Systems Analysis in Lilongwe. This revealed very high rates of TB amongst those presenting with chronic cough from the poorest areas of urban Lilongwe. For example in 2002 in Area 56, one of the poorest sub-districts of urban Lilongwe it was found that 25% of chronic cough cases tested positive as compared to 15% in less poor areas (Areas 18 and 49) [20]. This might, possibly, indicate that people presenting from Area 56 are already very sick, and have faced a longer pathway to accessing care for TB than their neighbours from Areas 18 and 49.

As highlighted earlier the gender ratio reveals that more men are diagnosed with TB compared with women although there are variations between districts. Qualitative studies conducted in selected districts have revealed gender differences in accessing resources for accessing care. For example, a study conducted in urban Lilongwe revealed intra/inter household differences in access to resources for seeking care[21]. Most women have to seek permission from their husbands to use resources for care seeking, which can lead to delay.

### Impact of care seeking and TB on patients and their households in Malawi

The social and economic consequences of tuberculosis not only affect the livelihood of patients, but also their households. In Malawi quantitative and qualitative studies were conducted under the TB Equity Project (which preceded REACH Trust), to analyse the impact of care seeking and TB illness on patients and their households. Stratified analysis was carried out using different indicators in different studies, including analysis by poverty status. The impact of TB on patients was analysed mainly by assessing the costs of TB before and after diagnosis. Costs of seeking care were classified as direct and indirect. Direct costs consisted of transport costs for the patient and guardian(s), food costs and any payments for diagnosis and treatment [22]. Indirect costs included number of working days lost due to illness, reduced income due to illness as well as loss of productivity in the medium term. The costs incurred were generally higher before diagnosis. One of the factors contributing to this is the fact that patients had to make several visits to health providers before diagnosis, as documented in the pathways to care section above. Most expenditure incurred was for transportation for both patients and their guardians.

Although aggregate costs for poor people in all studies tend to be lower in real terms than other social groups, costs relative to annual or monthly income are much higher for the poor than the non poor (Table 1).

**Table 1:** Total cost (in Kwacha) for a TB diagnosis in urban Malawi

	**All patients (urban)**	**Poor patients**	**Non-poor patients**
Number of respondents (% Total)	179	128 (72%)	51 (28%)
Total direct costs (MK)	942	798	1293
Total opportunity costs (MK)	1197	351	2170
Total costs (MK)	2139	1149	3463
Total costs as percentage of monthly income	134%	248%	124%
Total costs as percentage of monthly income after food expenditure	206%	584%	176%

US$1 = 75.7 Malawi Kwacha

Source: Kemp J.R, Mann G, Nhlema Simwaka B, Salaniponi FML, Squire S.B, Can Malawi's poor afford free TB services? Patient and household level costs associated with a TB diagnosis in Lilongwe. 2007. Bulletin of the World Health Organisation 85(8)

### The National TB Control Programme responses to equity challenges

This section highlights the ways in which the National TB Control Programme has tried to adapt its programme to better meet the needs of poor patients and guardians and to try and capture some of the estimated 52% of missing cases. These adaptations are three fold, and the first two aim to ease patients' journeys through the pathway to care. One set of initiatives attempts to increase poor patients' access to a TB diagnosis and hence intensify case finding. The second set of adaptations aims to ease the burdens patients face during treatment. The third set includes integration of TB and HIV related activities to enhance TB patients' access to HIV and AIDS treatment and care.

The REACH Trust in collaboration with the National TB Control Programme is undertaking operational research involving building partnerships with informal providers living close to communities to *enhance access to TB diagnosis*. One example of a community intervention is 'Extending Services to Communities; which is being pilot-tested in urban Lilongwe with funding from the Norwegian Association for Heart and Lung Patients. Through this intervention the Programme is working with storekeepers to equip them with advisory and referral skills to refer chronic cough cases and with community leaders to develop skills on health promotion. The proportion of Lilongwe city's total annual notifications of smear positive TB arising from the areas where the intervention was carried out rose significantly (Kauma from 0.2% to 1.3%, [p = 0.002], Ngwenya from 1.4% to 3.2%, [p = 0.004]) while the proportion reported from the control area did not rise significantly (Chinsapo from 2.7% to 3.3% [p = 0.44]) (Nhlema-Simwaka, 2007).

The standard DOTS strategy has been re-adapted in Malawi in order to ease the burdens *patients face whilst on treatment*. Initially patients had to stay in hospital for direct observation of pill taking during the intensive phase of treatment. Direct observation of pill taking has now been decentralised from health workers to guardians at the community level – either community representatives or family members [23]. This is arguably a pro-poor and patient centred intervention as it gives patients the choice of who should observe their treatment and avoids the costs and opportunity costs associated with long stays in hospital that can be particularly problematic for poor women and men. However, research has shown that caring for TB patients at home can bring economic and psychological stress to guardians, who are mainly female [24]. The NTP needs to work in partnership with carers and guardians at the community level to help ease some of these burdens.

Given high rates of TB and HIV co-infection other adaptations include *the integration of HIV/AIDS activities*. In response, the NTP developed HIV/AIDS activities which include routine voluntary counselling and testing (VCT) for TB, administration of cotrimoxazole to reduce the effect of opportunistic infections (OI) and referring TB patients for Antiretroviral Therapy ART within all districts [25]. Cross working and referral is arguably an equitable intervention as it increases the access of all TB patients to VCT, OI treatment and ART where appropriate. However the current monitoring and evaluation system for both TB and ART scale-up does not include data on number of cases cross-referred for both diagnosis and treatment between the two programmes. There is also need to document the challenges which poor and vulnerable TB patients face in accessing HIV/AIDS services, especially those from rural areas.

## Discussion

The absence of a prevalence survey severely curtails our epidemiological knowledge base on TB in Malawi. Prevalence surveys conducted in the Philippines and Hanoi, Vietnam revealed high numbers of missing cases from notification data. In the Filipino context the poor had high prevalence compared to rural settings [26]. In Hanoi, there was a greater disparity between the male to female ratio among notified cases compared to the prevalence male to female ratio, revealing under-notification of female cases [27]. A prevalence survey in Malawi would produce knowledge on prevalence by socio-economic status, gender, age and geography which could be used to tailor interventions to try and meet the needs of different groups.

In Malawi as in Nigeria, The Gambia, Vietnam and Burkina Faso, poverty and gender interplay to shape pathways to care seeking at both community and health systems levels [28-33]. Rural populations in particular face health systems related barriers as diagnosis depends on efficient transport and communication between rural health facilities and district hospitals In the Malawian context as in Thailand and India, the financial costs of TB treatment are very high, and for poor patients in particular, can lead to further spiralling into poverty[34,35].

The Malawian NTP has made some progressive steps to try and promote equity in access and adherence to TB in a context of extreme poverty and high levels of TB and HIV co-infection. However, given high levels of missing cases from case notification data there is need for further operational research, innovation and programme adaptation to further intensify case finding. These need to be geared to particular rural and urban Malawian contexts. In order to increase case detection the use of public-private partnerships have been explored in resource poor contexts, for example in India and Nepal [36,37].

Further work is needed in this area in Malawi, especially given severe human resource constraints in the formal health sector and the multiple barriers poor groups face in accessing formal health services. With a limited number of private for profit providers, especially in rural areas there is need to think creatively about what constitutes the private sector, looking particularly at further development of partnerships with community groups and a range of informal providers who live close to communities. Partnerships between the National TB Control Programme and traditional healers in South Africa and the Gambia have proved effective and could also be explored in Malawi [38,39]. The largest private-not-for-profit health provision in Malawi (mission-supported health facilities) is already integrated with public provision through the collaboration between Christian Health Association of Malawi (CHAM) and Ministry of Health.

Furthermore, simplification of the laboratory and diagnostic processes can also help to reduce the costs and opportunity costs poor patients face. There is a need to improve the speed and turnaround of results, and develop strategies to shorten the diagnostic pathway (technology used and numbers of smears submitted). The international literature shows that improved diagnostic efficiency can be achieved through examining a reduced number of specimens and accelerating the collection of samples to within a single day [40-42]. There is also a need to reconsider the need for rural patients to spend their first 2 weeks after TB diagnosis in hospital as this proves a significant barrier to many.

With the exception of Newell et al 2005 there has been limited discussion on either the programmatic or equity related opportunities and challenges disease control programmes face in moving from a vertical structure towards decentralisation and SWAps. For the Malawian NTP the move arguably provides a new impetus to strengthen relationships with district health officers, providers close to communities and community representatives. Strong relationships of this kind will provide a platform from which to jointly develop and implement district specific pro-poor and gender sensitive strategies to increases case detection and ease the pathway to care.

## Conclusion

The Malawi National Tuberculosis Control Programme is well established with a good reputation within the sub Saharan region. The programme has attempted to be responsive to the needs of different social groups through the development of community based activities to intensify case finding amongst poor groups. Given increasing TB notification due to HIV co-infection, high numbers of missing cases, and a context of poverty and gender inequity there is need for further programme adaptation, innovation and operational research. The new environment of the PoW, EHP and SWAp offers news opportunities for strengthening relationships with multiple players at district level and for developing responsive pro-poor and gender sensitive interventions to ease patients pathway to care. It is time to seize these opportunities.

## Competing interests

The author(s) declare that they have no competing interests.

## Authors' contributions

All authors read and approved the final manuscript. Specifically each author made the following contributions:

Bertha Nhlema Simwaka designed overall synthesis the study and undertook specific analysis on routine data.

George Bello contributed to the synthesis study and also carried out the routine data analysis for the TB and Poverty Network for Action Secretariat.

Rhehab Chimzizi and Hastings Banda contributed to the identification of resources for review, areas for consideration and write up of the paper.

Bertie Squire framed the questions, hypothesis and design of the equity analysis of routine TB data and was co-principal investigator, with Julia Kemp, on all the TB Equity and EQUI-TB Knowledge Programme studies quoted in the synthesis. He participated in writing and editing of the manuscript.

Sally Theobald in collaboration with Bertha Simwaka led the process of synthesis and facilitated the stakeholder workshop, contributed in the writing up of both the reports and academic paper for publication.
